# New Fossil Tingidae (Hemiptera: Heteroptera) from the Mid-Cretaceous of Myanmar, with Remarks on the Phylogenetic Relationships within the Family

**DOI:** 10.3390/insects12100887

**Published:** 2021-09-30

**Authors:** Yuxin Wang, Huiying Tang, Dong Ren, Yunzhi Yao

**Affiliations:** College of Life Sciences and Academy for Multidisciplinary Studies, Capital Normal University, Xisanhuanbeilu 105, Haidian District, Beijing 100048, China; 2190802038@cnu.edu.cn (Y.W.); thanghuiying@126.com (H.T.); rendong@cnu.edu.cn (D.R.)

**Keywords:** lace bugs, amber, new genus, parallel evolution

## Abstract

**Simple Summary:**

The members of the Tingidae family are generally known as “lace bugs” because of the lacelike network of areoles adorning the pronotum and hemelytra. The family is distributed worldwide and the earliest fossils of lace bugs are from the Early Cretaceous period. A total of 16 species in eight tingids genera have been described from the mid-Cretaceous Burmese (Kachin) amber of northern Myanmar. But the placement of six Cretaceous genera is still uncertain. In this paper, we describe a new genus and species *Latidorsum carinbifarium* Wang, Tang & Yao gen. et sp. nov., and present remarks on the phylogenetic relationships of Tingidae based on morphological features from extinct and extant genera clarifying the phylogenetic status of six species.

**Abstract:**

A new genus and species, *Latidorsum carinbifarium* Wang, Tang & Yao gen. et sp. nov., is assigned to Phatnomatini (Tinginae), which is described from the mid-Cretaceous Burmese amber. A parsimonious analysis based on 50 morphological characters with 26 terminals clarifies the phylogenetic status of the new fossils and examined relationships among the presently analysed genera of the family Tingidae. The resulting cladogram supporting Tingidae are reconfirmed as monophyletic and divided into four main clades, with relationships as follows: (Vianaidinae + ((*Burmacader multivenosus* + *Burmavianaida anomalocapitata*) + (*Sinaldocader rasnitsyni* + (*Gyaclavator kohlsi* + Tinginae + Cantacaderinae)))). Anterior length of bucculae distinctly projecting in front of head, sometimes in contact is not a synapomorphic character for Cantacaderinae, which evolves in parallel in Tinginae and Cantacaderinae.

## 1. Introduction

Tingidae, the so-called ‘lace bugs’, are of the order Cimicomorpha, comprising about 320 genera and 2600 species from all over the world, containing 45 genera with 76 species of the fossil record. They range in length from about two to eight mm, are phytophagous and host specific, with a few exceptions [1,2]. Drake and Ruhoff (1965) had divided Tingidae into three subfamilies, Vianaidinae, Cantacaderinae, and Tinginae. The Cantacaderinae were divided into two tribes, Cantacaderini and Phatnomatini; the Tinginae were divided into three tribes, Tingini, Litadeini, Ypsotingini [3]. This taxonomic scheme has been hitherto widely adopted, but several other research has provided additional systematic insights. In the past two decades, a consistent result of phylogenetic analysis for Tingidae has been obtained: Vianaidinae is the sister group to Cantacaderinae + Tinginae and Phatnomatini belongs in the Tinginae [4,5,6,7,8].

To date, there are eight genera with 16 species of Tingidae from mid-Cretaceous Burmese amber [8,9,10,11,12,13,14,15,16,17,18,19,20,21,22,23,24]. But the placement of six Cretaceous genera is still uncertain; they exhibit a combination of characters of the subfamilies or tribes [8,9,10,11,12,13,14,15,16,17].

The present paper describes a new Phatnomatini (Tinginae) genus and species, *Latidorsum carinbifarium* Wang, Tang & Yao gen. et sp. nov., from the mid-Cretaceous of Myanmar and present remarks on the phylogenetic relationships of Tingidae based on morphological features from extinct and extant genera. Many well-preserved amber specimens have been collected from the Hukawng Valley, Kachin State, in northern Myanmar, approximately 100 km southwest of the Village of Tanai [25,26]. The strata contain various arthropods, eight classes, 66 orders, 568 families, 1174 genera, 1738 species [24]. Although the geological age of Burmese amber previously has been controversial [27], a recent U–Pb dating of zircons have been established at 98.79 ± 0.62 Ma, equivalent to the earliest Cenomanian [28,29,30].

## 2. Materials and Methods

### 2.1. Materials and Terminology

Analyses were based on the examination of new amber materials stored in the Key Lab of Insect Evolution and Environmental Changes, College of Life Sciences, Capital Normal University, Beijing, China (CNU, Curator: Yunzhi Yao), and collected from Kachin (Hukawng Valley) of Northern Myanmar. The morphological study was performed by examining and photographing specimens under a Nikon SMZ25 microscope with an attached Nikon DS-Ri2 digital camera system. Morphological terminology mainly followed Drake & Ruhoff (1965) [3]. All measurements are in millimeters (mm).

### 2.2. Taxon Sampling and Character Choice

In accordance with the previously published phylogeny of Tingidae [4,5,7], we choose one species of Thaumastocoridae (*Xylastodoris luteolus* Barber, 1920) and two species of Miridae (*Onymocoris izzardi* Drake & Slater, 1957, *Myiomma kukai* Yasunaga & Hayashi, 2002) as our outgroup taxa. The 23 ingroups comprise 14 extant taxa and nine fossil taxa. All terminal taxa are species. For the extant species, the widespread and type-species of each genus were selected. We used the same set of characters coded by Wappler et al. (2015) [7], 50 characters were coded as unordered and with equal weight. The data matrix used in this phylogenetic analysis is presented in Table S1. Missing data were assigned as ‘?’ and inapplicable data as ‘-’.

### 2.3. Phylogenetic Analysis

The data matrix was constructed using Nexus Data Editor v0.5.0 [31] in File S1. Phylogenetic analysis was performed using maximum parsimony analysis in WINCLADA v1.00.08 [32] with NONA script [32,33]. The analysis was performed setting to keep 10,000 maximum trees, 1000 replications and 100 starting trees per replication, using WINCLADA software. A repeated analysis for verification was run in TNT v1.5 [34] in Figure S1 used ‘Traditional Search’, the minimum length was set to be found 100 times and Bremer support was calculated through the script ‘bremer.run’ of the program.

## 3. Results

### 3.1. Systematic Paleontology

Order Hemiptera Linnaeus, 1758.

Suborder Heteroptera Latreille, 1810.

Infraorder Cimicomorpha Leston, Pendergrast & Southwood, 1954.

Family Tingidae Laporte, 1832.

Subfamily Tinginae Laporte, 1832.

Tribe Phatnomatini Drake & Davis, 1960.

Genus *Latidorsum* Wang, Tang & Yao gen. nov.

**Figure 1:**
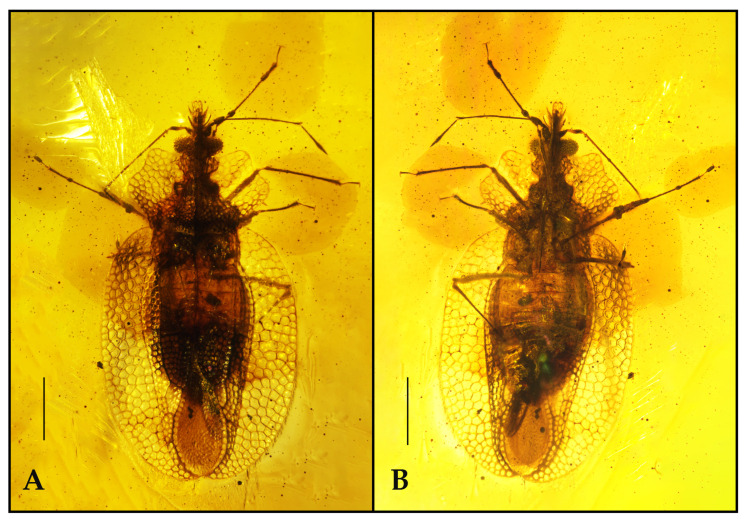
Habitus of *Latidorsum carinbifarium* gen. et sp. nov., holotype CNU-HET-MA2014010. (**A**) Photograph in dorsal view. (**B**) Photograph in ventral view. Scale bars = 0.5 mm.

Type species. *Latidorsum carinbifarium* sp. nov. (Figure 1).

Etymology. Named after its broad pronotum from the Latin word ‘latum’ (meaning wide) and dorsum. Gender feminine.

**Figure 2:**
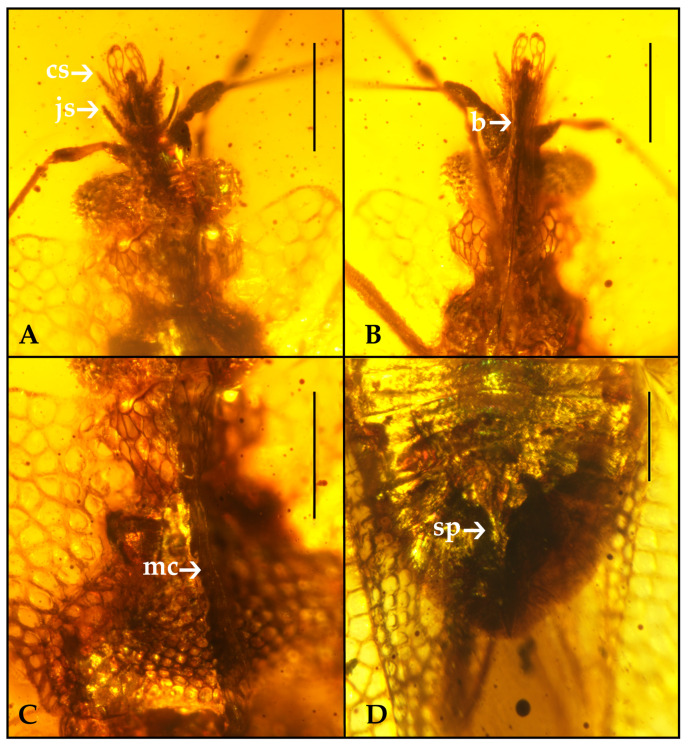
*Latidorsum carinbifarium* gen. et sp. nov. photographed with morphological details, holotype, CNU-HET-MA2014010. (**A**) head spines. (**B**) bucculae. (**C**) median carina. (**D**) female genital segment. Abbreviations: cs = clypeus, js = jugal spine, b = buccula, mc = median carina, sp = subgenital plate. Scale bars = 0.25 mm.

Diagnosis. Macropterous, body moderately oblong-oval. Head projecting anteriorly, with four spines, one pair of jugal, and one pair of clypeal spines (Figure 2A). Bucculae extended half-length of the head, converging and almost touching by their apices (Figure 2B). Antenna four-segmented, segment III longest, almost six times the length of segments I and II. Paranota broad, median carina elevated with one or two rows of areolae. Hemelytra stenocostal area absent, membrane without veins.

Remark. The habitus of our fossil is rather close to that of the genus *Tingiphatnoma* Guilbert & Heiss, 2019. Both genera have one pair of clypeal spines; antennae segment III longest; pronotum without a median triangular projection; median carina foliate and elevated; scutellum distinct; stenocostal area absent; membrane without veins. But our fossil differs from the latter because it has a long head surpassing the apex of the antennal segment I (a short head not surpassing antennal segment I in *Tingiphatnoma*); bucculae extended half-length of the head (the bucculae not extending in front of clypeus in *Tingiphatnoma*); paranota broad, with five or six rows of wide areolae and bilobate (vs. paranota extending in front into a spiny process and three areolae wide in *Tingiphatnoma*).

*Latidorsum carinbifarium* sp. nov. (Figure 1).

Holotype. CNU-HET-MA2014010. Adult female, well preserved.

Etymology. This species name is a combination of the Latin word ‘carin-’ (meaning carina) and ‘bifarius’ (meaning two rows), referring the median carina with two rows of areolae. The gender is feminine.

Locality and horizon. Lowermost Cenomanian, amber deposits from the Kachin State, Northern Myanmar.

Diagnosis. Head longer than wide, with long and curved spines in clypeal and in front of the antennal segment I. Rostrum long, four-segmented, reaching posterior margin of metasternum. Antennal segments III and IV with sparse pilosity and segment III thinner than first two segments, segment IV elongate spindle-shaped. Paranota with large areolae and bilobate, median carina extending from anterior margin of the collar to posterior margin of pronotum (Figure 2C). Scutellum exposed, small, triangular. Hemelytra distinctly subdivides by veins into costal, subcostal, discoidal, and sutural areas, cells of costal area larger than subcostal area, subcostal and discoidal areas subdivided by six and one transverse veinlets respectively.

#### Description

Head with long anteocular portion, 3.65 times as long as wide (head length measured from posterior margin of eyes to apex of clypeus), with four long, curved spines: one pair of jugal and a pair of clypeal spines. Bucculae produced in front of clypeus, extending half-length of the head, consisting of two rows of areolate, converging and almost touching by their apices. The eyes are convex, quite well projecting laterad. Antennae slender, about 2.95 times as long as width of head, antennae four-segmented, segments I and II shortest and thickest, first segment approximately the same length as second, segment III longest almost six times the length of segments I and II, and segment IV elongate spindle-shaped with sparse pilosity. Rostrum long, four-segmented, reaching posterior margin of metasternum.

Pronotum 2.24 times as wide as long with the areolate surface, the areolae smaller than those of paranota, pronotum with deeply sinuate lateral margins, posterior margin feebly convex without a median triangular projection. Collar areolae bigger than pronotum and inflated forming an inverted trapezoidal hood, partly covering the head, with a median foliate and raised carina with one or two rows of large subquadrate and rounded areolae extending from anterior margin of the collar to posterior margin of pronotum. Paranota broad, with five or six rows of wide areolae and bilobate, lateral margin slightly sinuate at middle and anterior, posterior margin slightly tilted upwards. Metathoracic scent gland orifice not visible. Rostral sulcus distinctly wide, not interrupted by two transverse carinae between meso- and metathorax. Mesosternal carinae slightly sinuate in the middle part, metasternal carinae very slightly elevated, forming very low oval cells and posteriorly a rounded transverse rib closing rostral sulcus. Scutellum exposed, small, triangular.

Hemelytra completely developed, extending far beyond abdomen beneath, hyaline, sharply widened at base, covered by areolae. Clavus wide, with four areolae at the widest part, apex reaching about half of the length of hemelytra, separated from mesocorium by a clear commissure. Costal area wide, with six rows of areolae at the widest part, the number of rows quickly diminishing backward up to five, preserved along most of costal area and near hemelytron apex number of rows of cells diminishing to three and in the very tip to one. Cells of costal area more or less tetragonal, pentagonal, and irregular angled and rounded form, larger than subcostal area. Subcostal area narrower than costal area, separated by six distinct transverse veinlets and located not fixed position in both hemelytra. Discoidal area slightly broader than subcostal area, hemelytron with one elevated transverse veins. R + M delimiting subcostal and discoidal areas slightly elevated and thick, fused with CuA at two thirds of the forewing. Sutural area narrow, one row of areolae wide. Stenocostal area absent. Membrane (zone of hemelytral overlap) not areolate; hind wings membranous.

Venter. Abdominal segments II and III fused; abdominal sternum VII with posterior medial projection (Figure 2D).

Legs. Long and slender, femora not incrassate, tibiae thin and cylindrical with sparse pilosity, tarsi two-segmented with curved claws, segment I shorter than II.

Dimensions. Body length 2.88; maximal width of body 1.51; length of head 0.41; length of antennal segments I–IV: 0.10, 0.12, 0.66, 0.30; length of rostrum segments I–IV: 0.07, 0.61, 0.27, 0.03; length of pronotum 0.49, width 1.1; length of hemelytron 1.91; length of fore leg: femur 0.51, tibia 0.66, tarsomeres I–II: 0.04, 0.09; length of mid leg: femur 0.64, tibia 0.72, tarsomeres I–II: 0.04, 0.09; length of hind leg: femur 0.54, tibia 0.65, tarsomeres I–II: 0.04, 0.09.

### 3.2. Phylogenetic Analysis

**Figure 3:**
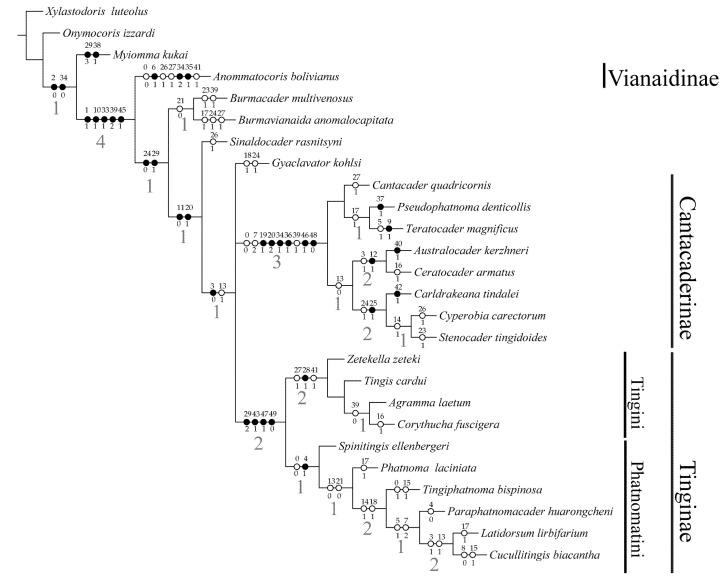
The strict consensus tree for the analysed Tingidae: tree length = 106 steps; consistency index = 0.59; retention index = 0.80. ●, non-homoplastic changes; ◯, homoplastic changes. The larger red (bold) numbers below the branches are Bremer support values.

The maximum parsimony analysis provides two equally parsimonious trees of 105 steps with a CI = 60 and a RI = 81. The strict consensus tree has 106 steps (CI = 59, RI = 80) and is shown in Figure 3, with unambiguous characters mapped. Bremer Supports are shown in Figure 3. The results of our phylogenetic analysis are as follows: Tingidae were reconfirmed as monophyletic based on five synapomorphies: ocelli absent (character 1:state 1), labial groove on thoracic sternum present (10:1), ostiole of metathoracic glands anterior on metepisternum (33:1), parempodia absent (39:2), female abdominal sternum VII with posterior medial projection (45:1). Tingidae were subdivided into four clades, Vianaidinae are considered to be the most plesiomorphic clade among extant and extinct lace bugs, and placed as sister to the remaining Tingidae, as proposed by Schuh et al. (2006) [5]. *Burmacader multivenosus* Heiss & Guilbert, 2013 + *Burmavianaida anomalocapitata* Souma, Yamamoto & Takahashi, 2021 are the sister group of the remaining Tingidae except *Anommatocoris bolivianus* Schuh, Cassis & Guilbert, 2006; *Sinaldocader rasnitsyni* Golub & Popov, 2012 is the sister-group of the *Gyaclavator kohlsi* Wappler et al., 2015 + Tinginae + Cantacaderinae. We consider *Burmacader multivenosus* + *Burmavianaida anomalocapitata* and *Sinaldocader rasnitsyni* as stem-group of *Gyaclavator kohlsi* + Tinginae + Cantacaderinae based on our result, but the relationships among the three species of two clades are weakly supported, this suggests that the phylogenetic status of stem-group needs further study. The strict consensus tree shows *Gyaclavator kohlsi* in a trichotomy with Tinginae and Cantacaderinae and this result is in agreement with Wappler et al. (2015) [7]. The Tinginae are formed by the tribe Phatnomatini and Tingini and they are sister groups. Our new amber species belongs to Phatnomatini.

## 4. Discussion

### 4.1. Clade Burmacader Multivenosus + Burmavianaida Anomalocapitata and Clade Sinaldocader Rasnitsyni

Supported the sister-group relationship of the *Burmacader multivenosus* + *Burmavianaida anomalocapitata* and others Tingidae except *Anommatocoris bolivianus* based on the following two synapomorphies: costal area broad, more than 2 areolae medially (24:0), hemelytra with small irregular areolae (29:1). *Sinaldocader rasnitsyni* are sister-group of the *Gyaclavator kohlsi* + Tinginae + Cantacaderinae on the basis of two synapomorphies: antennal segment II distinctly shorter than antennal segment III (11:0), the number of pronotal carinae one to three (20:1). *Burmacader multivenosus* + *Burmavianaida anomalocapitata* differ from the crown group of *Gyaclavator kohlsi* + Cantacaderinae + Tinginae by characters: spines on head absent (3:2); antennal segment II subequal to antennal segment III (11:1); the number of pronotal carinae, none (20:0). *Sinaldocader rasnitsyni* is distinguished from *Gyaclavator kohlsi* + Cantacaderinae + Tinginae by: spines on head absent (3:2); membrane in macropterous form normally developed (30:0). The above four characters were considered as synapomorphies of Cantacaderinae + Tinginae (Wappler et al., 2015) [7], so we think *Burmacader multivenosus* + *Burmavianaida anomalocapitata* and *Sinaldocader rasnitsyni* as stem-group of *Gyaclavator kohlsi* + Cantacaderinae + Tinginae based on these data. The results reaffirm the basal position of *Burmacader multivenosus* in Guilbert & Heiss (2019) results [8]. Additionally, our results do not support the treatment that *Sinaldocader rasnitsyni* is a member of the Cantacaderinae, as proposed by Popov & Golub (2019) [35].

### 4.2. Clade Gyaclavator Kohlsi + Tinginae+ Cantacaderinae

The monophyly of the clade *Gyaclavator kohlsi* + Tinginae + Cantacaderinae is supported by one synapomorphy: spines on head straight (3:0), this result is in agreement with Wappler et al. (2015) [7] based on the morphological evidence.

Cantacaderinae is monophyletic on the basis of six synapomorphies: lateral carinae on collar present (19:1), number of pronotal carinae five (20:2), scent gland peritreme developed as crevice-like (34:1), trochanters fused with femora (36:1), gonoplacs membranous (46:1), rudimentary spermatheca present (48:0). Anterior length of bucculae distinctly projecting in front of head, sometimes in contact (7:2), which are crucial for taxonomic identification in Cantacaderinae, and it is considered as the synapomorphies of subfamily Cantacaderinae by Wappler et al. (2015) [7]. The relationship of *Latidorsum carinbifarium* Wang, Tang & Yao gen. et sp. nov., *Paraphatnomacader huarongcheni* Guilbert & Heiss, 2019, *Cucullitings* *biacantha* Du & Yao, 2018 were uncertain because this character places them outside the known tribes and subfamilies. In addition, extant Cantacaderinae species both have this bucculae (7:2) and extant Tinginae not, but it presents in the new fossil Tinginae, so this character prone to a complex evolution. According to our analysis, this character is a homoplastic character for Cantacaderinae and the clade *Paraphatnomacader huarongcheni* + *Cucullitingis biacantha* + *Latidorsum carinbifarium*, which evolved in parallel in Tinginae + Cantacaderinae. So, the character is not a synapomorphies character for Cantacaderinae.

The Tinginae is also monophyletic and supported in this analysis by four apomorphic characters: hemelytra with larger areolae (29:2), abdomen (lateral sclerites) with a double set of lateral sclerites (43:1), pseudospermatheca present (47:1), vagina (genital chamber) small (49:0). The tribe Phatnomatini are as sister group to the Tingini, which are also monophyletic based on one synapomorphy: clypeal spine present (4:1). The tribe Tingini is also characterized by a single synapomorphy: clavus weakly developed and depressed below level of mesocorium (28:1). *Latidorsum carinbifarium* Wang, Tang & Yao gen. et sp. nov. has two synapomorphies: hemelytra with larger areolae (29:2), clypeal spine present (4:1), and an autapomorphic character: paranotum bilobed (17:1). Hence, we believe that *Latidorsum carinbifarium* should place in the Tinginae tribe Phatnomatini and be recognized as a new genus. The result also supported that *Tingiphatnoma bispinosa* Guilbert & Heiss, 2019, *Spinitings ellenbergeri* Heiss & Guilbert, 2013, *Paraphatnomacader huarongcheni*, *Cucullitings biacantha* are included in the Tinginae supported by one synapomorphic character and in tribe Phatnomatini by one synapomorphy as for *Latidorsum carinbifarium*, but the relationships among 6 genera need further study. The present analysis does not offer a well-supported phylogenetic classification within the Phatnomatini, because characters dealing with the shape of the scent gland peritreme, ventral and genital morphology are not visible and limit on the fossil species, and the fossil taxa are not representative in Phatnomatini. So, future studies would be to improve Phatnomatini phylogeny with a bigger taxa sample and more scent gland peritreme, ventral and genital morphology characters using modern documentation technics, such as micro-CT and electron microscope.

In addition, the oldest fossil record of Tingidae was *Archetingis ladinica* Montagna, Strada & Tintori, 2018 from the Middle Triassic of the Swiss side of Monte San Giorgio [36], but the identification of this poorly preserved specimen is doubtful [2] and in our view an areolate margin of the hemelytron more likely belongs to connexivum of abdomen based on the photograph and some reported features (i.e., antennae and eyes) in the line drawing not preserved in the fossil, so we treat *Archetingis ladinica* as of this doubtful placement.

## Data Availability

Data is contained within the article or supplementary material.

## Supplementary Materials

The following are available online at https://www.mdpi.com/article/10.3390/insects12100887/s1, Figure S1: the strict consensus tree of the most parsimonious tree calculated using TNT; Table S1: data matrix (.dox); File S1: Nexus file (nex).
