# Supplementary material for: New Fossil Tingidae (Hemiptera: Heteroptera) from the Mid-Cretaceous of Myanmar, with Remarks on the Phylogenetic Relationships within the Family
Source: Insects. 2021 Sep 30;12(10):887. doi: 10.3390/insects12100887 (PMC8540450; doi:10.3390/insects12100887)
Supplement: Supplementary file 1 [file insects-12-00887-s001.zip › Table S1, data matrix.pdf]

Table S1. Character matrix for extant and fossil Tingidae.

	0	1	2	3	4	5	6	7	8	9	10	11	12	13	14	15	16	17	18	19	20	21	22	23	24	25	26	27	28	29	30	31	32	33	34	35	36	37	38	39	40	41	42	43	44	45	46	47	48	49
Xylastodoris Luteolus	-	0	1	2	-	-	0	0	0	0	0	1	0	0	0	0	0	-	-	0	0	2	1	0	2	0	0	0	0	0	0	1	-	0	3	0	0	0	0	0	0	0	0	-	-	0	0	0	1	-
Onymocoris izzardi	-	0	1	2	-	-	0	0	0	0	0	1	0	0	0	0	0	-	-	0	0	2	0	0	2	0	0	0	0	0	0	1	-	0	3	0	0	0	0	0	0	0	0	-	-	0	0	0	1	-
Myiomma kukai	1	0	0	2	0	0	0	0	0	0	0	1	-	0	0	-	0	-	-	0	0	2	0	0	2	0	0	0	0	3	0	0	-	0	0	0	0	0	1	0	0	0	0	0	-	0	0	0	1	1
Anommatocoris bolivianus	0	1	0	2	0	0	1	0	1	0	1	1	0	0	0	-	0	-	-	0	0	2	1	0	2	0	1	1	0	0	0	1	?	1	2	1	0	0	0	2	0	1	0	0	2	1	0	?	1	1
Agramma laetum	1	1	0	0	0	0	0	1	1	0	1	0	0	1	0	0	0	0	0	0	1	1	-	0	0	0	0	1	1	2	1	-	0	1	0	0	0	0	0	0	0	1	0	1	1	1	0	1	1	0
Corythucha fuscigera	1	1	0	0	0	0	0	1	1	0	1	0	0	1	0	0	1	0	0	0	1	1	-	0	0	0	0	1	1	2	1	-	0	1	0	0	0	0	0	0	0	1	0	1	1	1	0	1	1	0
Tingis cardui	1	1	0	0	0	0	0	1	1	0	1	0	0	1	0	0	0	0	0	0	1	1	-	0	0	0	0	1	1	2	1	-	0	1	0	0	0	0	0	2	0	1	0	1	1	1	0	1	1	0
Zetekella zeteki	1	1	0	0	0	0	0	1	1	0	1	0	0	1	0	0	0	0	0	0	1	1	-	0	0	0	0	1	1	2	1	-	0	1	0	0	0	0	0	2	0	1	0	1	1	1	0	1	1	0
Phatnoma laciniata	0	1	0	0	1	0	0	1	1	0	1	0	0	0	0	0	0	1	0	0	1	0	-	0	0	0	0	0	0	2	1	-	0	1	0	0	0	0	0	2	0	0	0	1	1	1	0	1	1	0
Australocader kerzhneri	0	1	0	1	0	0	0	2	1	0	1	0	1	0	0	1	0	0	0	1	2	0	-	0	0	2	0	0	0	1	1	-	1	1	1	0	1	0	0	1	1	0	0	0	0	1	1	0	0	1
Cantacader quadricornis	0	1	0	0	0	0	0	2	1	0	1	0	0	1	0	0	0	0	0	1	2	1	-	0	0	2	0	1	0	1	1	-	1	1	1	0	1	0	0	1	0	0	0	0	0	1	1	0	0	1
Carldrakeana tindalei	0	1	0	0	0	0	0	2	1	0	1	0	0	0	0	0	0	0	0	1	2	0	-	0	1	1	0	0	0	1	1	-	1	1	1	0	1	0	0	1	0	0	1	0	0	1	1	0	0	1
Ceratocader armatus	0	1	0	1	0	0	0	2	1	0	1	0	1	0	0	2	1	0	0	1	2	0	-	0	0	2	0	0	0	1	1	-	1	1	1	0	1	0	0	1	0	0	0	0	0	1	1	0	0	1
Cyperobia carectorum	0	1	0	0	0	0	0	2	1	0	1	0	0	0	1	0	0	0	0	1	2	0	-	0	1	1	1	0	0	1	1	-	1	1	1	0	1	0	0	1	0	0	0	0	0	1	1	0	0	1
Pseudophatnoma denticollis	0	1	0	0	0	0	0	2	1	0	1	0	0	1	0	0	0	1	0	1	2	1	-	0	0	2	0	0	0	1	1	-	1	1	1	0	1	1	0	1	0	0	0	0	0	1	1	0	0	1

Stenocader tingidoides	0	1	0	0	0	0	0	2	1	0	1	0	0	0	1	0	0	0	0	1	2	0	-	1	1	1	0	0	0	1	1	-	1	1	1	0	1	0	0	1	0	0	0	0	0	1	1	0	0	1
Teratocader magnificus	0	1	0	0	0	1	0	2	1	1	1	0	0	1	0	0	0	1	0	1	2	1	-	0	0	2	0	0	0	1	1	-	1	1	1	0	1	0	0	1	0	0	0	0	0	1	1	0	0	1
Gyaclavator kohlsi	1	1	0	-	-	0	-	1	-	0	1	0	0	1	0	0	0	0	1	-	1	2	-	0	1	2	0	0	0	1	1	-	-	-	-	-	-	0	-	-	-	-	0	-	-	-	-	-	-	-
Cucullitingis biacantha	0	1	?	1	1	1	0	2	0	0	1	0	0	1	1	1	1	0	1	0	1	-	-	0	0	0	0	0	0	2	1	0	?	?	?	?	0	0	0	2	-	0	0	?	-	1	-	-	-	-
Burmacader multivenosus	1	1	?	2	0	-	?	0	0	0	1	1	0	0	1	0	0	0	0	0	0	0	-	1	0	0	0	0	0	1	1	0	?	?	?	?	0	0	0	1	-	0	0	?	-	1	-	-	-	-
Paraphatnomacader huarongcheni	0	1	?	0	0	1	0	2	?	0	1	0	0	0	1	0	0	0	1	0	1	0	-	0	0	0	?	1	0	2	1	0	?	?	?	?	0	0	0	?	-	-	0	?	?	?	-	-	-	-
Tingiphatnoma bispinosa	1	1	?	0	1	0	0	1	?	0	1	0	0	0	1	1	1	0	1	0	1	?	-	0	0	0	?	1	0	2	1	0	?	?	?	?	0	0	0	?	-	-	0	?	-	?	-	-	-	-
Spinitingis ellenbergeri	0	1	?	0	1	?	?	1	?	0	1	0	0	1	?	0	0	?	?	0	1	1	-	0	0	0	?	0	?	2	1	0	?	?	?	?	0	0	0	?	-	?	?	?	-	?	-	-	-	-
Sinaldocader rasnitsyni	?	1	?	2	0	-	?	?	?	0	?	0	0	0	1	0	0	0	0	0	1	2	-	0	0	0	1	0	0	1	0	-	?	?	?	?	-	0	-	-	-	-	0	-	-	-	-	-	-	-
Burmavianaida anomalocapitata	1	1	0	2	0	-	0	?	?	0	1	1	0	0	1	0	0	1	0	0	0	0	-	0	1	0	?	1	0	1	1	0	?	?	?	?	?	0	0	2	0	?	0	?	-	-	-	-	-	-
Latidorsum lirbifarium	0	1	?	1	1	1	0	2	1	0	1	0	0	1	1	0	1	1	1	0	1	-	-	0	0	0	0	0	0	2	1	0	?	?	?	?	0	0	0	2	-	0	0	1	-	1	-	-	-	-
