# Supplementary figures and images for: New Fossil Tingidae (Hemiptera: Heteroptera) from the Mid-Cretaceous of Myanmar, with Remarks on the Phylogenetic Relationships within the Family
Source: Insects. 2021 Sep 30;12(10):887. doi: 10.3390/insects12100887 (PMC8540450; doi:10.3390/insects12100887)

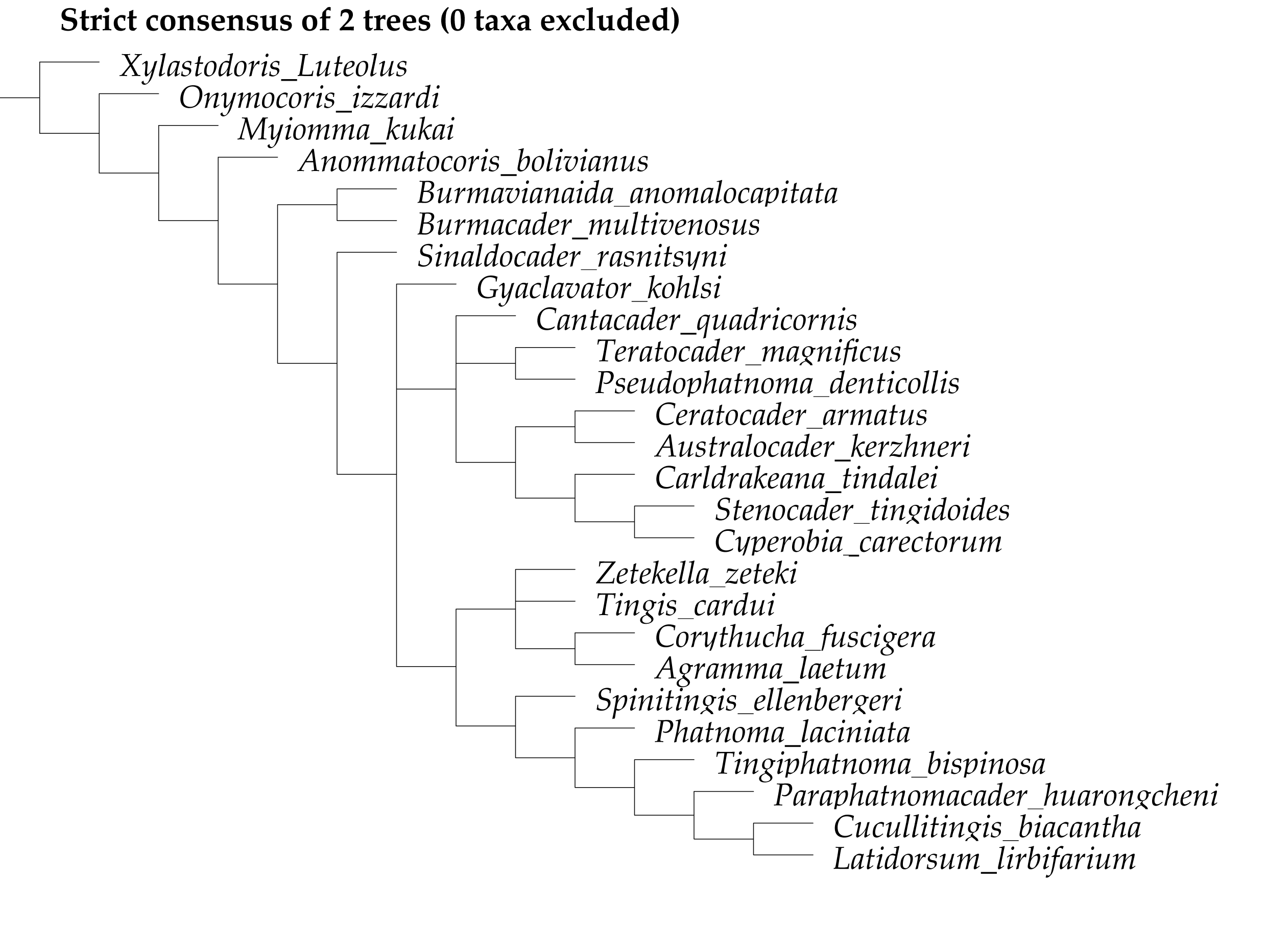

Supplement: Supplementary file 1 [file insects-12-00887-s001.zip › Figure S1, the strict consensus tree of the most parsimonious tree calculated using TNT.tif]
